# Problem gambling and support preferences among Finnish prisoners: a pilot study in an adult correctional population

**DOI:** 10.1108/IJPH-07-2018-0041

**Published:** 2019-12-05

**Authors:** Kalle Lind, Anne H. Salonen, Johanna Järvinen-Tassopoulos, Hannu Alho, Sari Castrén

**Affiliations:** 1 Alcohol, Drugs and Addictions Unit, Terveyden ja hyvinvoinnin laitos, Helsinki, Finland; 2 Alcohol, Drugs and Addiction Unit, Terveyden ja hyvinvoinnin laitos, Helsinki, Finland; 3 Terveystieteiden tiedekunta, Itä-Suomen yliopisto, Kuopio, Finland; 4 Faculty of Social Sciences, University of Helsinki, Helsinki, Finland; 5 Institute of Clinical Medicine, Helsingin Yliopisto, Helsinki, Finland; 6 Alcohol Drugs and Addictions Unit, National Institute for Health and Welfare, Helsinki, Finland; 7 Department of Psychology and Speech and Language Pathology, University of Turku, Turku, Finland; 8 Helsingin Yliopisto, Helsinki, Finland

**Keywords:** Prisoners, Quantitative research, Substance use, Problem gambling, Addiction treatment, Criminal behaviour

## Abstract

**Purpose:**

The purpose of this paper is to explore the prevalence of potential problem gambling among Finnish prisoners; the associations between problem gambling and demographics, substance use and crime-related factors; and problem gamblers’ support preferences.

**Design/methodology/approach:**

Prisoners (*n*=96) from two Finnish prisons were recruited between December 2017 and January 2018. The estimated response rate was 31 percent. Gambling problems were measured using the Brief Biosocial Gambling Screen. The participants were asked to report their gambling both for one year prior to their incarceration and for the past year. The independent variables were demographics (age, gender and marital status), substance use (alcohol, smoking and narcotics) and crime-related factors (crime type, prison type and previous sentence). Statistical significance (*p*) was determined using Fischer’s exact test.

**Findings:**

Past-year pre-conviction problem gambling prevalence was 16.3 percent and past-year prevalence 15 percent. Age, gender, smoking, alcohol or illicit drug use were not associated with past-year problem gambling before sentencing. One-third of the prisoners (33.3 percent) who were sentenced for a property crime, financial crime or robbery were problem gamblers. One-quarter (24 percent) of all participants showed an interest in receiving support by identifying one or more support preferences. The most preferred type of support was group support in its all forms.

**Research limitations/implications:**

It is recommended that correctional institutions undertake systematic screening for potential problem gambling, and implement tailored intervention programs for inmates with gambling problems.

**Originality/value:**

This study provides a deeper understanding of problem gambling in prisons. Problem gambling is associated with crime and also seems to be linked with serving a previous sentence. Early detection and tailored interventions for problem gambling may help to reduce reoffending rates.

## Introduction

It is estimated that problem gambling is five to ten times higher in the adult correctional population than in the general population (Turner *et al.*, 2013, 2017; Riley and Oakes, 2015; Williams *et al.*, 2005; May-Chahal *et al.*, 2017). In Finland, population surveys indicate that 3.3 percent (The South Oaks Gambling Screen, SOGS=3+, Lesieur and Blume, 1987) of people suffer from problem gambling (Salonen and Raisamo, 2015); which falls in the middle of the suggested worldwide problem gambling range of 0.12–5.8 percent (past 12 months) reported by Calado and Griffiths (2016). Problem gambling is an important criminogenic factor, with the majority of identified inmates with problem gambling having committed a problem gambling-related criminal offense (Turner *et al.*, 2009; Brown, 1987; Meyer and Stadler, 1999; Laursen *et al.*, 2016). The types of crime committed by these offenders, such as fraud, forgery, embezzlement, larceny, selling drugs or stolen goods, shoplifting, burglary and petty theft or robbery, are specifically aimed at covering gambling losses and at making continued gambling possible (Turner *et al.*, 2009; Lind *et al.*, 2015; Kuoppamäki *et al.*, 2014; Abbott and McKenna, 2005; Abbott *et al.*, 2005; Potenza *et al.*, 2000). A higher percentage of problem gamblers than social gamblers commit illegal acts in order to finance their gambling habit or to pay off gambling debts (Reith and The Scottish Centre for Social Research, 2006).

In Finland, there is a scarcity of research concerned with prisoners; no published data is currently available on problem gambling in the prison population. It is almost ten years since the last major prisoner health study, but while this research did address mental health and substance dependence, problem gambling was excluded (Joukamaa *et al.*, 2010). The current study is the first peer-reviewed published report on the prevalence of problem gambling among Finnish prisoners. Gambling and problem gambling among prisoners is an important area of research for various reasons. Among prisoners, undetected and untreated problem gambling is widely viewed as a risk to community re-entry and can lead to re-incarceration. Problem gambling may also have a damaging impact on significant others (Salonen *et al.*, 2016) and victims of gambling-related crimes, which can make re-entry difficult. Problem gambling associated with criminal behavior requires rigorous interventions and clear policies to reduce the incidence or re-occurrence of problematic gambling and to ease the burden on the criminal justice system.

In order to create an effective support mechanism for problem gambling prisoners, it is important to understand the demographics and comorbidities of prison populations. High rates of problem gambling are closely linked with being a young man, minority group status and comorbidities such as substance abuse (Crockford and el-Guebaly, 1998), but problem gambling also occurs among women inmates (Riley *et al.*, 2017; Williams *et al.*, 2005; Abbott and McKenna, 2005).

Problem gambling often remains undetected and undiagnosed, and it is less automatically assessed than substance abuse and mental health problems (Turner *et al.*, 2017; Brooks and Blaszczynski, 2011; Williams *et al.*, 2005). Problem gambling is often seen as a marginal issue that does not warrant the same attention as more visible problems such as substance use, especially when personnel resources are limited. The integration of interventions into broad addiction support programs such as Counselling, Assessment Referral Advice and Throughcare in the UK, a low threshold low/medium intensity, non-clinical drug treatment service for prisoners (Offender Health Research Network, 2010), involves multiple challenges. Shame and the fear of stigma, not wanting to quit gambling and lack of awareness about available support or help are major barriers to seeking help among both the general population and inmates (Suurvali *et al.*, 2008; Turner *et al.*, 2017). On the other hand, it has been reported that inmates with severe gambling problems do tend to seek help (Riley *et al.*, 2017). The first step to making progress is, therefore, to identify the links between problem gambling and other life-control problems and then to integrate problem gambling treatment with prisons’ substance abuse treatment (Obstbaum *et al.*, 2016) or broader prison rehabilitation programs. This would guarantee the best possible support services and treatment paths for this particular target population (Turner *et al.*, 2017). Identifying the problem and providing sufficient services are crucial to the goal of preventing crime and reducing reoffending rates (Meyer and Stadler, 1999).

The empirical part of this study is based on a questionnaire among prisoners and probationers, the aim of which was to assess the prevalence of problem gambling, substance use and gambling behavior related to crime, as well as prisoners’ and probationers’ support preferences with regard to problem gambling. A further purpose is to discuss possible support and treatment options.

### Aims

In order to build more effective support systems for prisoners, we need a more in-depth understanding of how substance use and problem gambling are associated and how their concomitant presence can be addressed in correctional institutions. This study set out to explore the prevalence of problem gambling among Finnish prisoners by gender; the association between potential problem gambling, age of gambling and problem gambling onset, and demographics (age, gender and marital status), substance use (alcohol, smoking and narcotics) and crime-related factors (crime type, prison type and previous sentence); and to explore the support preferences of those prisoners willing to seek help for their problem gambling.

## Methods

### Setting

The Criminal Sanctions Agency in Finland runs 26 prisons, which include both closed and open institutions. The two prisons selected for this survey represent two different types of penitentiaries. Both are located in the criminal sanctions region of Western Finland. Turku prison is a closed high-security prison with a capacity to house 255 inmates. Vanaja prison is an open prison and has two units, one for women and one for men. Open prisons are often the last step of a prison sentence before inmates make the transition back to regular life. Vanaja open prison also has a family ward where inmates can stay with their small children.

The data for this study were collected in these two prisons between December 2017 and January 2018. Before data collection, the researchers met with staff at both prisons, providing information about the purpose of the study and instructions regarding data collection. Staff members distributed the questionnaires to the participants, who also received an information sheet about the study and its purpose. All the data were collected by prison guards as the researchers did not have access to the wards. The researchers visited the prisons personally to promote the study and discuss the preferred and most appropriate method of data collection with the heads of the prisons. Based on these discussions the decision was made to organize data collection via the guards, who were provided with written instructions and who handed out the questionnaires, information sheets and informed consent forms to prisoners. The timing of data collection was based on the prisons’ own routines. No prior advertisements or notifications were issued about the study in the prisons. The questionnaires were collected in sealed ballot boxes to ensure confidentiality and to demonstrate that prison staff did not have access to the data.

In addition to prisoners, our survey included persons in supervised probationary freedom. Detainees were excluded as they had not yet been sentenced. The estimated response rate is calculated using statistics provided by the Criminal Sanctions Agency: the number of prisoners changes daily, which means it is difficult to give the precise number of prisoners reached in our study. On January 1, 2018 Turku had 194 prisoners belonging to our target group and Vanaja 59 prisoners. In all we received 96 responses from 312 prisoners (based on January 1 statistics), giving a response rate of 30.8 percent. The response rate was higher in Vanaja (66.1 percent) than in Turku (29.4 percent).

#### Measures

##### Problem gambling

the questionnaire instructions defined gambling as “games that are played for money.” Gambling problems were measured using the Brief Biosocial Gambling Screen (BBGS, Gebauer *et al.*, 2010), a three-item scale measuring neuro-adaptation, psychosocial characteristics and adverse social consequences of gambling (Table I). Based on their “yes” or “no” responses (with total scores ranging from 0 to 3), the participants were defined as potential problem gamblers if they scored one or more. In addition, participants who had answered “yes” to any of the BBGS items were instructed to answer two further questions concerning age of gambling onset and age of problem gambling onset. BBGS was originally developed to measure gambling problems in the past 12 months. Since gambling opportunities are scarce in prison settings, we asked the participants to fill out two different versions of BBGS: the first one inquired about gambling during the past 12 months before the moment of completing the questionnaire, and the second one concerning the 12 months before the start of their prison sentence (on the street).

##### Substance use

alcohol consumption was measured by using a modified version of the Alcohol Use Disorders Identification Test (AUDIT-C) (Bush *et al.*, 1998). AUDIT-C is a three-item screen used to identify persons who are hazardous drinkers or who have active alcohol use disorders (including alcohol abuse or dependence). It is based on a five-point Likert scale as follows: *a*=0 point, *b*=1 point, *c*=2 points, *d*=3 points *e*=4 points. In this study, total scores were counted by summing up the points for each item, and cut-off points recommended by Seppä (2010) were used to identify risky drinking among men (score⩾6) and women (score⩾5). Smoking was ascertained with the following yes/no question: “Have you smoked any of the following products: cigarettes, pipe, cigar or electronic cigarettes during the past year?” Lifetime illicit drug use was ascertained with the question: “Have you used narcotic substances?,” with a yes/no response option.

##### Crime-related variables

respondents were also asked to identify the primary crime for which they were currently sentenced. The specified crime types were: robbery; theft or property crime; murder, manslaughter or attempted murder; other violent crime; tax offense, false accounting or other financial crime; drug offense; driving under the influence; and other crime. In addition, the respondents were asked (yes/no) whether they had any previous sentences (Do you have previous sentences?). Finally, the participants were asked to say whether or not their current sentence was gambling related (Is your current sentence related to gambling?). Prison type was also included in the analysis.

##### Support preferences

the participants’ support preferences were assessed by listing different types of support options (see Table IV). Respondents were allowed to choose as many options as they wanted.

##### Demographics

background variables included gender (man/woman/other/do not want to disclose), age and current marital status (married or in a registered relationship/cohabiting/single/divorced/widowed).

##### Ethics

the study was conducted in accordance with the ethical standards of the Declaration of Helsinki. The Ethics Committee of the National Institute for Health and Welfare, Finland, approved the research protocol (THL/1701/6.02.01/2017). Additional approval was obtained from the Criminal Sanctions Agency. Potential participants received written and verbal information about the study and the principles of voluntary participation.

##### Data analysis

the data were analyzed using SPSS 23 software (SPSS, Inc., Chicago, IL, USA). Statistical significance (*p*) was determined using *χ*^2^ test (>2 groups) or Fisher’s exact test (2 groups): *χ*^2^ test was used for categorical variables when the test assumptions were valid and Fisher’s exact test was used when any expected cell count was less than five for a 2×2 table.

## Results

### Description of the participants

We had 96 prisoner participants, of whom 57.3 percent were men and 40.6 percent women (Table II). Just under one-third were aged 25–34 years, slightly over one-quarter were 35–44 years and one-quarter were 45–54 years. Among women the largest age group (41.0 percent) was 45–54 years, among men the largest age group (33.3 percent) was 25–34 years. Women were most often married or in a registered relationship (36.8 percent), among men the most common marital status was single (38.2 percent).

More than half (53.8 percent) of the participants used alcohol at a risky level (men 69.0 percent; women 31.0 percent). The Cronbach’s *α* for AUDIT-C was 0.747 (Table II). In the closed prison, 59.6 percent of the participants used alcohol at a risky level, compared to 39.3 percent in the open prison. Over 80 percent of the participants had smoked (men 90.9 percent; women 74.4 percent) at least once during the past 12 months. Overall, 37.0 percent of the participants (47.4 percent of men and 23.1 percent of women) had used narcotic substances in their lifetime.

Almost half of the participants reported that their principal offense was a violent crime (Table II). Almost one-third (31.9 percent) had been sentenced for murder, manslaughter or attempted murder. Violent crime was the most frequent category of crime among both men and women: 35.9 percent of women and 28.8 percent of men had been sentenced for murder, manslaughter or attempted murder. One-quarter of women and less than one-tenth of men had been sentenced for a drug offense; 5.2 percent of women and 21.2 percent of men for robbery, theft or property crime; and 10.3 percent of women and 7.7 percent of men for a tax offense, false accounting or other financial crime. Driving under the influence was the principal crime for 9.2 percent of men and 1.6 percent of women. Nearly 60 percent of the participants reported that they had been sentenced previously (39.5 percent of women and 73.6 percent of men). More than half (59.6 percent) of the respondents were in a closed high-security prison. These participants were predominantly men (89.1 percent), whereas women accounted for the bulk of the open prison inmates (82.1 percent).

### Problem gambling

In total, 16.3 percent of the participants (18.4 percent of men and 14.8 percent of women) indicated having a gambling problem during the 12 months prior to their incarceration (BBGS=1+) (Table I). The Cronbach’s *α* value for BBGS was 0.747. Psychosocial characteristics (12.9 percent) were the most commonly recognized criterion of problem gambling, followed by neuro-adaptation (12.0 percent) and adverse social consequences criteria (10.6 percent). Among men, the most often endorsed criterion was neuro-adaptation (11.1 percent), which refers to becoming irritable or anxious when trying to stop gambling. Among women, the most common criterion was psychosocial, referring to problems in trying to keep family or friends from knowing about their gambling (18.4 percent). Overall, women gave more positive (yes) responses to all three items of the BBGS questionnaire (10.5 percent) than men (5.6 percent) when evaluating past-year gambling, but due to the low count data, no formal statistical test was performed on group differences.

In addition, 15 percent of the participants were identified as potential problem gamblers (BBGS=1+) during the past year. 92.9 percent of them also scored at least one point for the 12 months before incarceration. Similarly, 86.7 percent of those who were identified as potential problem gamblers pre-incarceration also scored at least one point for past-year BBGS. Mean age of gambling onset was 14.73 (SD=5.78), which corresponded with the relatively early age of problem gambling onset (mean=22.45, SD=8.73).

### Problem gambling and correlates

The proportion of problem gamblers was highest in the age group 35–44 years, regardless of gender. The most common marital status for problem gamblers was single (47 percent). Among those with risky alcohol consumption, 14.3 percent also had a gambling problem. In addition, 20 percent of the participants with a history of drug use and 17.5 percent of those who smoked presented with gambling problems. Prison type (closed/open) was not associated with problem gambling prevalence (Table III).

One-third of those who had been sentenced for an income-generating crime had a gambling problem. Tax offenses, false accounting and other financial crimes were the most common reasons for being sentenced among problem gamblers, followed by drug offenses and property crimes. There was a statistically significant association (*p*=0.012) between crime type and problem gambling: problem gambling was more common among those who were sentenced for property crime, financial crime or theft. Among those who had a previous sentence, 24.1 percent (18 percent of men and 40 percent of women) can furthermore be defined as problem gamblers. Among women, having a previous sentence had a statistically significant (*p*=0.011) association with gambling problems.

Among the six participants whose principal offense was gambling related, five were potential problem gamblers. There was an association (*p*<0.000) between problem gambling and gambling-related crime. Of those six inmates whose principal offense was gambling related, four had been sentenced for a property offense, financial crime or robbery.

### Support preferences

One-quarter (24 percent, *n*=23) of the participants showed an interest in receiving support by identifying one or more support preferences (Table IV). There were more participants who wanted support than those who were identified as potential problem gamblers in either BBGS (16.7 percent, *n*=16). The most preferred type of support was group support in all its forms, followed by personal discussion with a prison employee. Men in particular seemed to prefer group-based support and face-to-face discussions over other support types. Virtual support was more popular among women and, overall, women seemed to be more open to different types of support. Those whose principal offense was gambling related preferred personal conversations with a prison employee, mixed group support and a guided online forum.

## Discussion

### Prevalence

Prior to incarceration, past-year prevalence of potential problem gambling among the inmates of the two Finnish prisons surveyed was 16.3 percent. Our results therefore support previous studies indicating that the prevalence of problem gambling is higher in the criminal justice population than the general population (Turner *et al.*, 2013, 2017; Riley and Oakes, 2015; Williams *et al.*, 2005; May-Chahal *et al.*, 2017). In Germany, 7.5 percent of male and 3.6 percent of female prisoners were diagnosed as problem gamblers (Zurhold *et al.*, 2014).

Our sample can be compared against the general prison population in Finland based on statistics from the Criminal Sanctions Agency. The mean age of all prisoners in the country in 2017 was 37.3 years. Most of them were sentenced for a violent crime (40 percent) and one quarter for a property crime. Eight percent of prisoners in Finland were women (Criminal Sanctions Agency, 2017). Our results showed no statistically significant gender differences in problem gambling prevalence. Castrén *et al.* (2015) have earlier reported the same result for patients receiving opioid substitution treatment. Some studies indicate that men gamble more often and suffer from more severe problem gambling than women in the criminal justice population (Wallisch and Kerber, 2001; Kerber *et al.*, 2001), but others have found a higher gambling prevalence rate for female prisoners (Abbott and McKenna, 2005; Abbott *et al.*, 2005).

This finding of no gender differences may indicate a growing trend for women’s problem gambling (Salonen *et al.*, 2017; Romild *et al.*, 2016) or other confounding factors. If the problem gambling rate among women is nearing the same level as among men in general, gender-specific approaches will be required for prevention and treatment at the population level as well as among prisoners, where the aim is to reduce levels of recidivism (Riley *et al.*, 2017).

In this study, the most endorsed BBGS item was the psychosocial criterion, which refers to the consequences of problem gambling for social relationships. Problem gambling impacts significant others as well (Salonen *et al.*, 2016), and in some cases can even lead to intimate partner violence (Roberts *et al.*, 2016; Afifi *et al.*, 2010; Liao, 2008). The second most endorsed criterion of neuro-adaptation refers to the behavioral manifestations of withdrawal, and was reported more often by men than women. On the other hand, women reported psychosocial characteristics more often than men. This finding must be interpreted with caution because of our small sample size, but it certainly warrants further investigation of the different gender trajectories. Previous studies have shown that the onset of problem gambling among women is usually associated with stressful life situations and coping difficulties, traumatic experiences in childhood or later life and financial difficulties (Järvinen-Tassopoulos, 2016).

Problem gambling prevalence rates were quite similar for both timeframes, i.e. 12 months before incarceration and the previous 12 months. This might indicate that gambling problems are persistent and long lasting among prisoners. Measurement of the prevalence of problem gambling among prisoners involves several challenges. Future studies should collect data from incoming prisoners in order to avoid problems stemming from recall bias, which may be compounded by the prison setting and the different lengths of sentences. In the current study our focus was to assess the situation of those who were currently in prison, their preferences for support and to help develop practices of support in prisons, regardless of the length of sentence. Among our prisoners, age at problem gambling onset was lower than reported in previous prison studies (Turner *et al.*, 2009) and among help-seeking gamblers (e.g. Teo *et al.*, 2007). Age at gambling onset was also lower than in the general population (Salonen and Raisamo, 2015). Previous studies confirm that early gambling onset not only predicts the development of later gambling problems, but also mental health problems and substance abuse (Burge *et al.*, 2006). In adolescents, particularly males, problem gambling seems to be associated with various problem behaviors, such as substance use, violence and delinquency (Vitaro *et al.*, 2004; Winters *et al.*, 2002): individuals who are prone to one problem behavior are also more vulnerable to others. Similarly, antisocial and risk-taking behavior is a risk factor for problem gambling among adolescents (Dowling *et al.*, 2017; Stinchfield, 2000; Gupta *et al.*, 2006).

### Comorbidities

Alcohol risk consumption, smoking and narcotics use was very common in this prison population. Due to several limitations with regard to the measures used and their timeframes, however, these results must be considered tentative, even though they are closely in line with earlier findings (Fazel *et al.*, 2017). Although we found no significant association between problem gambling and other substance use, it is obvious that these problems do tend to accumulate among prisoners. It is well-established that substance abusers are overrepresented in prison populations (Fazel *et al.*, 2006; Lintonen *et al.*, 2011). Even though it is estimated that substance abuse is ten times more prevalent than in the general population (Joukamaa *et al.*, 2010), substance problems are not always detected in Finnish prison settings. In this sample, prisoners in a closed prison setting reported using alcohol at a risky level more often than those in an open prison setting. Future studies into problem gambling and the use of any substances among prisoners should use interviews alongside self-report questionnaires in order to ensure the reliability of the results.

Globally, most prisoners tend to come from the lower end of the socioeconomic spectrum; they have a low education and a wide range of physical and mental health problems. Since the 1980s, mental health problems have become increasingly common and better recognized among prisoners, leading to a growing recognition of the need for preventive measures and treatment options (Obstbaum-Federley, 2017; Joukamaa *et al.*, 2010). Indeed, it is crucial that comorbidities and depth of pathology are properly recognized before prognosis and treatment mechanisms are set up. Based on a pathways model of gambling, there is a possibility that prisoners may fall into the third subgroup of pathological gamblers, which is characterized by signs suggestive of neurological (Young *et al.*, 2015; Morde *et al.*, 2011) and neurochemical dysfunction, impulsivity and antisocial personality disorder (Blaszczynski and Nower, 2002; Nower and Blaszczynski, 2016). Future research could examine if problem gamblers, perhaps even by gender, in prison populations are in fact more likely to fall into the third subgroup. Both the accumulation of various problems and comorbidities suggest that this specific subgroup would greatly benefit from thorough clinical assessment.

### Gambling and crime

One-third of the participants who were identified as problem gamblers reported that their current sentence was gambling related. Similarly, Turner *et al.* (2009) reported that 65 percent of the prisoners studied in Canada with serious gambling problems were sentenced for a gambling-related offense. In New Zealand, 19 percent of female and 9 percent of male prisoners who were recently sentenced had a gambling-related offense (Abbott and McKenna, 2005; Abbott *et al.*, 2005). Despite the high rate observed by Turner, treatment for problem gambling is still not systematically integrated into prisoner health care anywhere in the world. There is clearly a need for preventive and supportive interventions and the early identification of problem gambling.

Problem gambling has many adverse consequences, one of which is criminal behavior. Problem gambling and crime can also be part of a risk-taking lifestyle (Mishra *et al.*, 2011). Previous studies indicate that problem gambling tends to accumulate in socio-economically vulnerable populations. As the spiral of the gambler deepens, there are ever fewer legal options to finance gambling. Eventually, severe financial difficulties and indebtedness can lead to property crimes. This study suggests that problem gambling is relatively common, especially among prisoners sentenced for financial crimes.

In line with previous research, the results of this study suggest that problem gamblers’ sentences are often associated with gambling, particularly with income-generating crime (see also Riley *et al.*, 2017; Riley and Oakes, 2015; Turner *et al.*, 2009; Abbott and McKenna, 2005; Abbott *et al.*, 2005). This is not surprising, since the aim and purpose of gambling-related crimes are precisely to finance gambling or to pay off gambling debts (Turner *et al.*, 2009; Lind *et al.*, 2015). In our study, one participant was sentenced for a violent crime other than homicide and one for a drug-related crime. Such gambling-related violent crimes may include domestic violence, debt collecting induced by problem gambling or laundering drug money by gambling.

Women who had a previous sentence were more likely to have a gambling problem (cf. Bevan and Wehipeihana, 2015). In order to reduce recidivism, it is important to identify and provide appropriate treatment for possible gambling problems as early as possible. Riley *et al.* (2017) reported that women prisoners’ help-seeking rate was higher than in the general population. As this seems to be the case, courts could encourage help seeking. Cuadrado and Lieberman (2012), for their part, recommend screening programs in view of the high proportion of problem gamblers among prisoners and the fact that they are charged with more severe type of crimes. In fact, it would be prudent to screen all offenders who enter the criminal justice system (e.g. court) to identify those in need of help as early as possible, for example, using a court diversion program (see Riley *et al.*, 2018). Future research could examine the prevalence of problem gambling among offenders entering the justice system.

### Support preferences

One-quarter (24 percent) of our participants showed an interest in receiving support by selecting one or more support preferences, which is in line with a previous study showing similar rates in prior help-seeking behavior (Riley *et al.*, 2017). The most preferred type of support was group support in all its forms, followed by personal discussions with a prison employee. The low proportion of prisoners willing to seek help may reflect the barrier that continues to deter people from seeking help (Turner *et al.*, 2017; Riley, Larsen, Battersby and Harvey, 2018; Riley, Baigent, Harris, Larsen, Nye and Battersby, 2018), or the individual’s motivational stage. One unique discovery in our results was the finding that there were more responses indicating preferred forms of help than possible problem gamblers. This may suggest the presence of hidden problems, but on the other hand, also that if the preferred type of help and support were readily available, the number of prisoners taking advantage would also be higher. This is crucial information for purposes of planning and tailoring interventions in correctional settings. Despite the relatively high prevalence of problem gambling among prisoners, this remains an understudied and underdiagnosed phenomenon. As Turner *et al.* (2017) note, one major issue is the lack of knowledge in the judicial system: unlike substance addictions, problem gambling is still seen primarily as a moral issue, something that is more of a bad choice rather than a true addiction. This is despite the fact that in the fifth edition of the *Diagnostic and Statistical Manual of Mental Disorders* (American Psychiatric Association, 2013), pathological gambling was renamed as a gambling disorder and moved from the category of impulse control disorder to non-substance related behavioral addiction. Problem gambling is surrounded by a negative social stigma and it might, therefore, be very difficult for outsiders to recognize the problem and for individuals to admit to the problem. The screening and early detection of possible problem gamblers among incoming prisoners is crucial to the effective prevention of recidivism, since untreated gambling problems coupled with accumulating debts can hamper and complicate rehabilitation into society. Financial desperation can greatly narrow the options available to problem gamblers.

Most treatment programs reviewed by Turner *et al.* (2017) take a biopsychosocial approach to problem gambling, using cognitive-behavioral therapy in group settings, with some programs focusing on prevention and others on treatment. Most problem gambling interventions in prison settings are integrated as part of general addiction treatment, while only few programs are specifically designed to address problem gambling. We still do not know what type of programs are most effective, since most treatment approaches used in prisons have not been evaluated. Evaluation is thus an important priority for the future. Prisons have limited personnel resources for gambling-related harm prevention, reduction and treatment, and substance addictions are considered a more visible problem than problem gambling. These kinds of factors may explain why substance addiction treatment is given priority over problem gambling.

### Limitations

This study was explorative in nature, investigating prisoners problem gambling in Finland. Although our results are in line with previous studies (Turner *et al.*, 2009; May-Chahal *et al.*, 2017; Cuadrado and Lieberman, 2012), they must be interpreted with caution due to the following limitations. Our sample was small and may not be representative of the broader prison population in Finland. The participation rate was low, which in part at least can be explained by the novelty of the research approach. Furthermore, we only received the total number of prisoners reached, and therefore we could not estimate response rates by gender. The cross-sectional nature of our study prevents any suggestion of causal associations or temporal relations. We did not inquire into the length of the participants’ sentences. Response bias cannot be excluded. Those who have been in prison for several years might not recall their gambling behavior before incarceration. Therefore, to avoid this problem, future studies should use a lifetime prevalence timeframe (Riley *et al.*, 2017). Our study shows no positive associations with other addictions, which is unusual. This is likely explained by the measures used, such as the specification of the illicit drugs (Babor *et al.*, 2010) used, the timeframe (current) AUDIT-C and the other limitations mentioned above.

Previous problem gambling surveys in the criminal justice population have used a variety of instruments, such as the South Oaks Gambling Screen and the Canadian Problem Gambling Severity Index (e.g. Turner *et al.*, 2009; May-Chahal *et al.*, 2017), the Lie-Bet (Zurhold *et al.*, 2014; Cuadrado and Lieberman, 2012) which limits the comparability of our results. To our knowledge, BBGS has not been used previously among prison populations. Furthermore, the timeframe of evaluations has varied from current to the past 6 or 12 months before incarceration through to lifetime (Abbott and McKenna, 2005; Turner *et al.*, 2009; Zurhold *et al.*, 2014; Lahn, 2005). In this study, we opted to use the BBGS due to its strong psychometric properties (Gebauer *et al.*, 2010) and its brevity. This brevity probably means that our figures for the prevalence of problem gambling are higher than those based on SOGS and PGSI or clinical evaluations. Future studies should therefore evaluate gambling severity using longer measures coupled with clinical assessments. Ours is the first study to use a brief screen of problem gambling in a prison setting and, at the same time, to inquire about support preferences among those who might be in need of support. We found no gender differences in problem gambling, which may have to do with the sample size or prison setting. This is an area that certainly warrants further investigation, as noted by others (Riley *et al.*, 2017). Overall, although indicative only, our results provide valuable insights for the research community, developers and decision makers alike.

### Implications

In 2016, the Criminal Sanctions Agency’s Health Care Unit was renamed as the Prisoners’ Health Care Unit and placed under the supervision of the National Institute for Health and Welfare. The purpose of this administrative reorganization was to integrate prisoners’ health care more closely with wider health care services and to improve the monitoring of health care provision. It will guarantee that prisoners have the same rights to health care as the general population, as discussed by Turner *et al.* (2017). In order to ensure that this basic requirement is met, it is necessary to have mechanisms in place for the early detection of problems, to increase general awareness of gambling problems, and to provide clear intervention guidelines for prison staff. As problem gambling often remains undetected and prison workers are trained and motivated to help (Tourunen and Kaskela, 2014; Turner *et al.*, 2017) and to look for signs of risky behaviors, prisons are a potentially important environment for effective intervention. The reception of the results of our pilot study was very positive at all levels of the Criminal Sanctions Agency. The next steps will be to increase policy makers’ awareness of gambling problems; to apply for funding for both quantitative and qualitative studies in prison settings; to draw up guidelines for assessment, support and treatment and to assess the efficacy of these guidelines in the future.

## Conclusions

Our study indicates that problem gambling is relatively common among prisoners and that they clearly need support. Surprisingly, despite the strong evidence provided by other studies, we found no association between problem gambling and other addictions. It is more common among inmates sentenced for a property crime, financial crime or robbery than those sentenced for violent crime other crimes. Among women, a previous sentence is associated with having a gambling problem. Based on the results, it is recommended that steps are taken to develop early detection systems and to make tailored treatment options more readily available. In addition to prisons, we also encourage courts to screen for at-risk and problem gambling and to promote help seeking in an effort to divert suitable offenders from incarceration to rehabilitation.

## Figures and Tables

**Table I:** Criteria of problem gambling as endorsed by prisoners (*n*=96) by gender

		All	Men	Women
Criteria	Question	*n* (%)	*n* (%)	*n* (%)
1. Neuro-adaptation	“During the 12 months before being convicted, did you become restless, irritable or anxious when trying to stop/cut down on gambling?”	11 (12.0)	6 (11.1)	5 (13.2)
2. Psychosocial characteristics	“During the 12 months before being convicted, did have you try to keep your family or friends from knowing how much you gambled?”	12 (12.9)	5 (9.1)	7 (18.4)
3. Adverse social consequences of gambling	“During the 12 months before being convicted, did you have such financial trouble as a result of your gambling that you had to get help with living expenses from family, friends or welfare?”	10 (10.6)	4 (7.3)	6 (15.4)
Problem gambling^a^ 12 months before conviction		15 (16.3)	8 (14.8)	7 (18.4)

**Notes:** BBGS, Brief Biosocial Gambling Screen, with yes and no response options. ^a^One or more positive responses (yes) to questions 1–3 indicated potential problem gambling during the 12 months before conviction

**Table II:** Participants’ demographics, substance use and crime-related factors

	All		Men		Women	
	*n*=96		*n*=55		*n*=39	
	*n*	%	*n*	%	*n*	%
*Gender*						
Men	55	57.3	–	–	–	–
Women	39	40.6	–	–	–	–
Other/missing	2	2.1	–	–	–	–
*Age group*						
18−24 years	7	7.5	6	11.1	1	2.6
25−34 years	28	30.1	18	33.3	10	25.6
35−44 years	25	26.9	16	29.6	9	23.1
45−54 years	23	24.7	7	13.0	16	41.0
55 years or more	10	10.8	7	13.0	3	7.7
*Marital status*						
Married or in a registered relationship	23	24.7	9	16.4	14	36.8
Cohabitation	22	23.7	13	23.6	9	23.7
Single	30	32.3	21	38.2	9	23.7
Divorced	16	17.2	10	18.2	6	15.8
Widowed	2	2.2	2	3.6	0	0.0
Alcohol risk consumption, yes	42	53.8	29	69.0	13	31.0
Tobacco smoking, yes	79	84.0	50	90.9	29	74.4
Use of narcotics, yes	34	37.0	25	47.2	9	23.1
*Crime type*						
Robbery	5	5.5	4	7.7	1	2.6
Theft or property crime	8	8.8	7	13.5	1	2.6
Murder, manslaughter or attempted murder	29	31.9	15	28.8	14	35.9
Other violent crime	15	16.5	9	17.3	6	15.4
Tax offense, false accounting, other financial crime	8	8.8	4	7.7	4	10.3
Drug offense	14	15.4	4	7.7	10	25.6
Drunken driving	6	6.6	5	9.6	1	2.6
Other crime	6	6.6	4	7.7	2	5.1
Previous sentence, yes	54	59.3	39	73.6	15	39.5
*Prison type*						
Closed high-security prison	56	59.6	49	89.1	7	17.9
Open prison	38	40.4	6	10.9	32	82.1

**Table III:** Association between the demographic factors and pre-conviction gambling problems

	Gambling problem^a^ *n* (%)	Significance
*Gender*		0.776
Men	8 (14.8)	
Women	7 (18.4)	
*Age*		0.527
18−34 years	6 (17.1)	–
35 years or more	9 (15.5)	
*Marital status*		0.463
Married, registered relationship or cohabitation	8 (17.8)	
Single, divorced or widowed	7 (14.9)	
*Alcohol risk consumption*^*a*^		0.388^c^
Yes	6 (14.3)	
No	3 (8.1)	
*Smoking*		0.456^c^
Yes	14 (17.5)	
No	1 (7.1)	
*Use of narcotics*		0.563
Yes	7 (20.0)	
No	8 (14.0)	
*Crime type*		0.012*
Property crime, financial crime, robbery	7 (33.3)	
Violent crime, drug offense or other crime	7 (10.0)	
*Previous sentence*		0.022*^c^
Yes	13 (24.1)	
No	2 (5.4)	
*Sentence related to gambling*		0.000**^c^
Yes	5 (83.3)	
No	10 (11.4)	
*Prison type*		0.971
Open prison	6 (15.8)	
Closed high-security prison	9 (16.1)	

**Notes:** *n*=96, ^a^BBGS=1+, Brief Biosocial Gambling Screen: one or more positive responses (yes) indicated potential past-year gambling problems; ^b^AUDIT-C risky drinking defined among men score⩾6 points and women score⩾5 points; significance is determined by Fischer’s exact test (two groups); ^c^expected cell count 5 or less. *p⩾0.05; **p⩾0.001

**Table IV:** Support preferences of problem gambling prisoners wanting help by gender

	All		Men		Women	
	*n*=22		*n*=15		*n*=7	
	*n*	%	*n*	%	*n*	%
Personal discussion with prison employee	10	45,5	6	60.0	4	40.0
Group support	17	77,3	12	75.0	4	25.0
Male or female group	11	50,0	7	63.6	4	36.3
Mixed group	7	31,8	4	57.1	3	42.9
Not specified	2	9,1	2	100.0	0	0.0
Telephone supported virtual treatment program	4	18,2	0	0.0	4	100.0
Supportive telephone discussions with a professional	5	22,7	1	20.0	4	80.0
Supportive telephone discussions with a peer	6	27,3	2	33.3	4	66.7
Guided discussion forum or other virtual help from outside the prison	7	31,8	3	42.9	4	57.1
